# Re-envisioning the Concept of Styalgia and Styloidectomy

**DOI:** 10.1055/s-0046-1817133

**Published:** 2026-05-08

**Authors:** Kavya Sivapuram, Prakash Bhadravathi Ganesh, Ambale Rudrappa Babu, Sandhya Dharmarajan, Bharathi M. B., Sandeep Shetty

**Affiliations:** 1 Department of Ear, Nose, and Throat, Mamata Academy of Medical Sciences, Hyderabad, Telangana, India; 2 Department of Ear, Nose, and Throat (ENT) – Head and Neck Surgery, JSS Medical College and Hospital (JSSAHER), Mysuru, Karnataka, India

**Keywords:** elongated styloid process syndrome, Eagle's syndrome, elongated styloid process, styloid-stylohyoid syndrome

## Abstract

**Introduction:**

Prevalence of elongated styloid process (SP) is 4% and only 4–10% of these patients are symptomatic. In this study morphology was assessed among the patients unlike the dry skulls. Comparison of elongation and angulation of SP between the case and control groups, as well as symptomatic correlation was done. This study also imparts information regarding symptomatic outcomes following medical and surgical management.

**Objective:**

To compare the anatomical variations in SP morphology and symptomatology in patients with and without stylalgia.

**Methods:**

A prospective study was done over a period of 1.5 years on 84 participants. The required details were recorded. Radio-imaging was done through Towne's and lateral view of skull X-rays to measure length, as well as medial and anterior angulations. Pain assessment was done with the visual analogue scale before and after conservative or surgical managements.

**Results:**

Mean age of participants was 37.2 ± 8.8 years, with a female preponderance. The most common symptom was recurrent oropharyngeal pain (67.8%). Elongated SP was observed in the case group with a mean length of 4.07 cm, compared with 2.36 cm in controls. The mean medial angulation of the SP was of 21.28° in the case group and of 16.77° in the controls. Anterior angulation in the case group was of 32.19°, compared with 21.74° in the controls. A statistically significant difference was noted in in length, medial and anterior angulations in the case group when compared with controls.

**Conclusion:**

Symptomatic elongated SP is a complex condition for many patients. Its precise identification will aid in early recovery from symptoms. Surgical management showed better symptomatic outcomes.

**Level of Evidence:**

Level 3.

## Introduction

The styloid process (SP) is a cylindrical, slender bony structure that projects from the inferior aspect of petrous part of the temporal bone. It lies antero-medial to stylomastoid foramen and offers attachment to 2 ligaments and 3 muscles namely stylohyoid and stylomandibular ligaments and stylohyoid, stylopharyngeus, styloglossus muscles forming the Riolan's bouquet. 1 2 It is derived from the Greek word *stylos* which means ‘pillar.’ 3 The SP is surrounded by various vital structures like glossopharyngeal, vagus, accessory nerves, internal jugular vein, internal carotid artery and its branches on medial aspect, facial and hypoglossal nerves, as well as external carotid artery on its lateral aspect. 1 4 5

The length of the SP is not consistent across all individuals, with studies reporting average lengths anywhere from 1.52 to 6 cm. 5 6 7 Prevalence of an elongated SP is estimated at around 4% of the general population and is more common in women than men. 5 6 7 8 9 This elongation of SP or calcification of stylohyoid apparatus (SP, stylohyoid ligament and lesser cornu of hyoid bone) is called Eagle's syndrome. Only 4 to 10% of the patients with elongated SP present with symptoms as per the data from previous studies. 1 2 10 11 This symptomatology of abnormal length of SP was named as stylalgia by Eagle in 1937. 10 Stylalgia patients present with nonspecific symptoms like recurrent pain in the oropharynx, chronic neck pain, dysphagia, foreign body sensation in throat, referred otalgia, tooth ache, headache, restriction of cervical movements. 6 8 9 12 Therefore, it is to be considered in differential diagnosis of patient presenting with chronic orofacial and cervical pain.

Elongated SP can be diagnosed by clinical examination and radiological evaluation. Its clinical diagnosis is made by palpating the tonsillar fossa. Radiological diagnosis is made by X-ray Towne's view, X-ray lateral view of skull, 6 12 and three-dimensional (3D) computed tomography (CT) evaluation. 3 7 Eagle reported the normal length of SP as around 2.5 cm, and any length beyond 3 cm is considered as elongated SP. 10

To date, various studies are done on anatomical variations of the SP on dry skulls, where the abnormal morphology was analyzed. 5 8 11 This study is peculiar as the morphology was assessed among the patients unlike the dry skulls and also includes a good sample size in comparison to other studies. The main aim of the study is to compare the elongation and angulation of SP between the patients presenting with symptoms of stylalgia and a control group and to correlate the symptoms with the elongation and abnormal SP angulation. This study also imparts information regarding the symptomatic outcome following medical and surgical management and emphasizes the need for considering Eagle's syndrome as a differential diagnosis in patients with chronic orofacial and neck pain.

## Methods

A prospective study was conducted over a period of 18 months. A total of 84 participants were divided into two groups. Group 1 included 42 patients with stylalgia symptoms meeting the inclusion and exclusion criteria. As a control group, 42 patients between 18 and 50-years-old, with any trauma to the ear and/or chronic otitis media without stylalgia symptoms were considered.

Patients between 18 and 50-years-old presenting to the Ear, Nose, and Throat (ENT) Outpatient Department (OPD) with stylalgia symptoms (recurrent pain in the oropharynx, chronic neck pain, dysphagia, foreign body sensation in throat, referred otalgia, tooth ache, headache, restriction of cervical movements).

The exclusion criteria were previous history of tonsillectomy, trauma in the region of hyoid complex, and/or cervical spondylosis. Proven cases of malignancy of oropharynx were also excluded.

Patient details, clinical and radiological findings were recorded. Complaints, past history, and relevant clinical examination findings in patients presenting with stylalgia (cases) were recorded. Radiological examination was done through Towne's and lateral view of skull X-rays in both cases and controls to measure length, medial and anterior angulation, as well as distance between two SPs. The X-ray imaging for all the cases and controls was done by the same person to avoid differences in position and technique, which can result in erroneous values. The person performing the measurements was blinded to each patient's cohort.

**Fig. 1:**
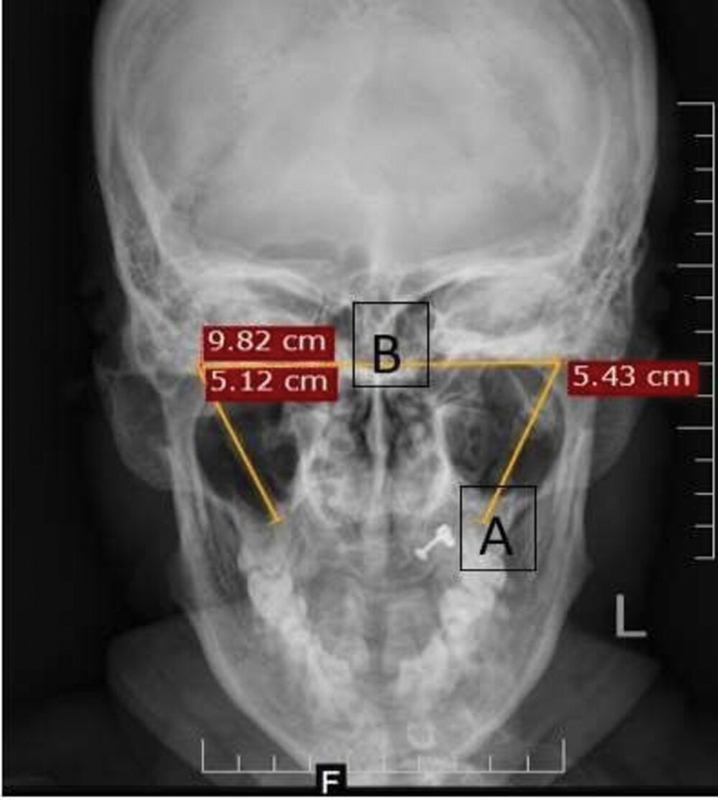
Radiograph of the styloid process (SP): ( **A** ) length; ( **B** ) distance between two SPs.

In the SP X-ray, Towne's view was used for measuring length and the distance between two SPs at its base. Length was measured from the cranial base up to the osseous tip of each SP ( Fig. 1A ). The distance between the two SPs was measured by a horizontal line drawn from the base of right-side SP to the base of the left-side SP ( Fig. 1B ).

**Fig. 2:**
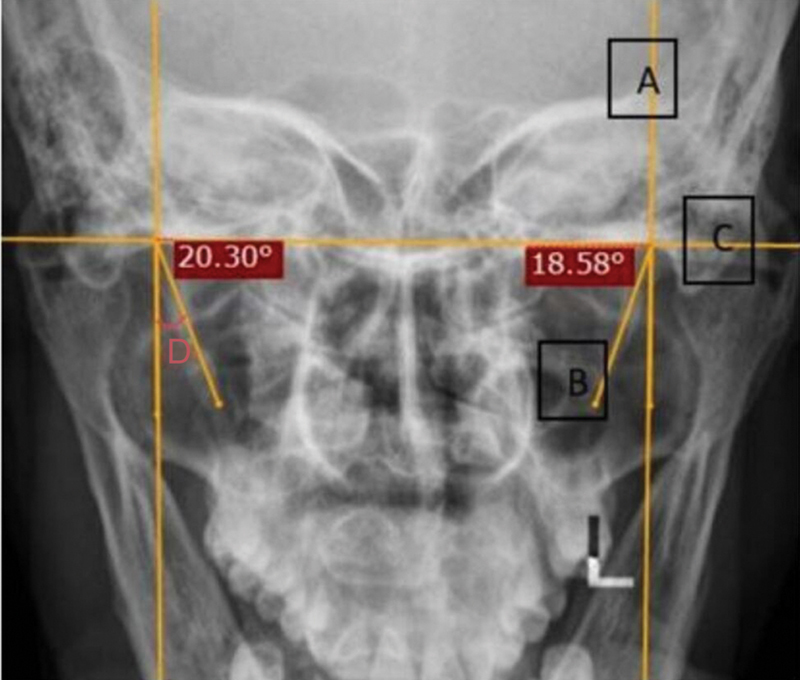
Median angulation of SP: ( **A** ) line through the base of the SP; ( **B** ) line through the body of the SP; ( **C** ) line from the bases of both SPd; and ( **D** ) median angulation.

Medial angulation was also measured on Towne's view. A vertical line was drawn passing through the cranial base of the SP ( Fig. 2A ). Another line was drawn passing through its body ( Fig. 2B ). A horizontal line was drawn connecting the bases of both, which was perpendicular to the first line ( Fig. 2C ). The measured angle between A and B gave the SP's medial angulation ( Fig. 2D ).

**Fig. 3:**
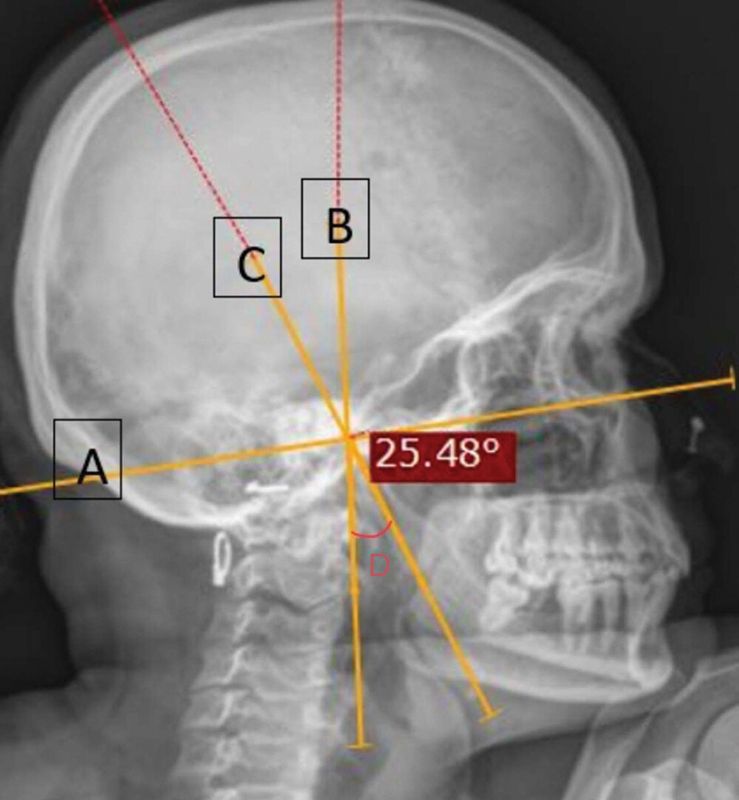
Anterior angulation of the SP: ( **A** ) Frankfort plane; ( **B** ) Line through the base of the SP; ( **C** ) line through the body of the SP; and ( **D** ) anterior angulation.

A true lateral projection using X-ray, in which the beam travels laterally with 0 degrees of angulation, was used to measure the SP's anterior angulation. The initial requirement for measuring this anterior angulation is establishing a Frankfort plane. This is obtained by drawing a line from the superior border of the external auditory canal to the lower border of the orbital rim ( Fig. 3A ). Another line is drawn passing through the SP's base ( Fig. 3B ). The angle is obtained between this vertical line and the one drawn passing through the SP's body ( Fig. 3C ) given the anterior angulation of the SP ( Fig. 3D ).

Once the diagnosis was confirmed, patients were given information regarding both conservative and surgical management options. One tablet of pregabalin 75 mg/day at night, amitriptyline 10 mg/day, one tablet at night for a period of 3 weeks, and tramadol hydrochloride one tablet in the morning for 1 week were given.

The main surgical management option was styloidectomy for patients who had persisting symptoms even after conservative treatment. Informed consent was obtained from all patients prior to the procedure. Surgical management was not performed in patients who did not give consent for the same. Pain assessment was done using the visual analogue scale (VAS) before and after conservative and surgical managements. Patients returned for follow-up at 3 weeks and 3 months.

### Statistical Analysis

A statistical analysis was performed by means of proportions for the categorical/binary variables, as well as mean, median, , and interquartile range (IQR) values for the continuous variables. Inferential statistics were done using the Chi squared or Fisher's exact tests, independent *t* -test, and Pearson's correlation. A *p* -value < 0.05 was considered statistically significant. The analysis was performed using the IBM SPSS Statistics for Windows (IBM Corp.) software, version 21.0.

## Results

A total of 84 participants were included in the present study. They were distributed into two groups, with 1: stylalgia cases, and 2: controls – Participants without stylalgia symptoms. The mean age of the participants was 37.2 ± 8.8 years in group 1 and 36.1 ± 8.3 years in group 2. There was a female preponderance, with male to female ratio of 1:2.2, without statistical significance ( *p* = 0.49). The average duration of symptoms was 9.2 ± 8 months.

**Fig. 4:**
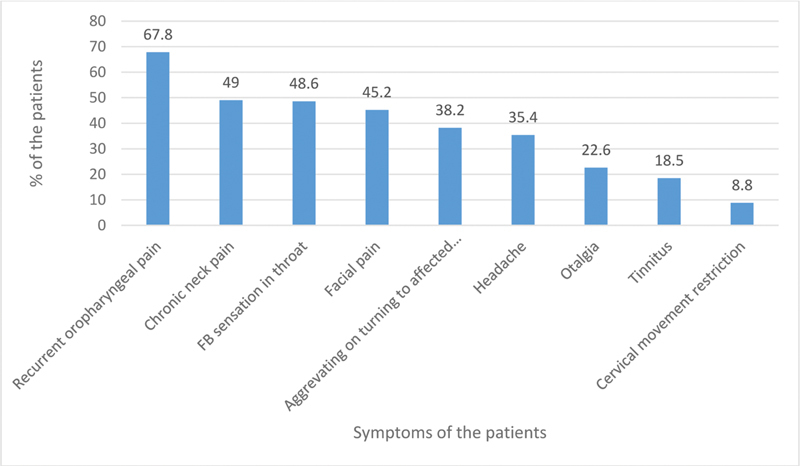
Percentage of complaints presented by the case group.

The most common presenting symptom was recurrent oropharynx pain (67.8%) Other complaints are shown in Fig. 4 . A majority (91%) of patients presented with unilateral symptoms (either right or left side), while 9% had bilateral symptoms. On clinical examination, elongated SP was palpable in 66.7% of patients.

The range, mean, and SD of length, as well as medial and anterior angulations of both cases and controls are shown in Table 1 . There was a statistically significant difference in the length, medial and anterior angulation among both groups. The length of SP was similar on both sides.

**Table 1:** Length, medial and anterior angulations of the styloid process in cases and controls on the right and left sides

Styloid process							*p* -value
	Case group			Control group			
	Range	Mean		Range	Mean		
Length: right (cm)	3.1–5.8	4.07 ± 0.64		1.7–3.1	2.36 ± 0.46		< 0.0001
Length: left (cm)	3.2–5.6	4.02 ± 0.80		1.8–2.9	2.28 ± 0.46		< 0.0001
Medial angulation: right	16.2–28.6°	21.28 ± 4.02°		12.4–24.2°	16.77 ± 2.94°		< 0.0001
Medial angulation: left	14.8–28.2°	20.6 ± 4.15°		12.8–23.7°	16.91 ± 3.47°		< 0.0001
Anterior angulation: right	24.6–49.4°	32.19 ± 5.85°		15.6–29.5°	21.74 ± 4.51°		< 0.0001
Anterior angulation: left	22.8–52.3°	33.66 ± 7.14°		13.8–28.4°	20.95 ± 4.19°		< 0.0001

**Fig. 5:**
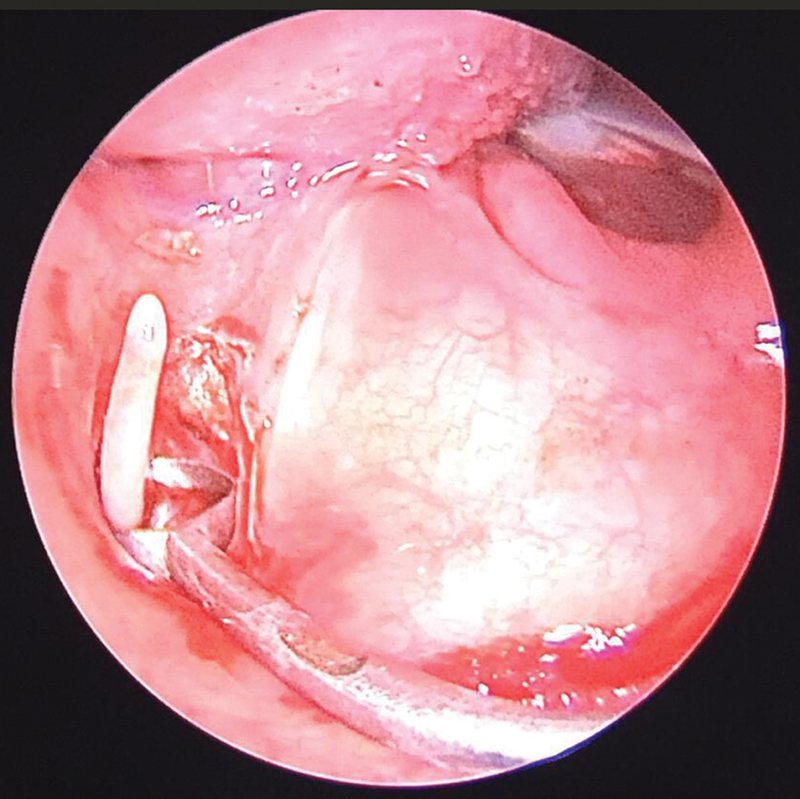
Intraoperative images of extracapsular styloidectomy.

The distance between both SPs in group 1 was 9.10 ± 0.57 cm and in group 2 it was 9.40 ± 0.43 cm, with no statistical significance ( *p* = 0.009). The distance between both SPs was measured to evaluate spatial anatomical differences, which can contribute to symptom development. Once the diagnosis was made, patients were counseled regarding conservative and surgical managements. Styloidectomy was done in the 26 patients who consented ( Fig. 5 ).

**Fig. 6:**
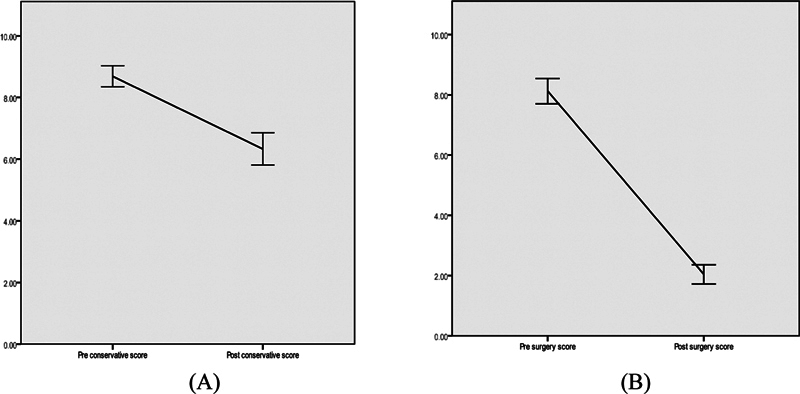
Graphical representation of the improvement in Visual Analogue Scale scores in ( **A** ) the conservative and ( **B** ) surgical managements.

The pre- and posttreatment scores for conservative and surgical options after 3 weeks are depicted in Fig. 6 , with a statistically significant difference. However, patients had more relief in symptoms with surgical management (presurgical: 8.12 ± 0.99; postsurgical at 3 weeks: 2.04 ± 0.75) compared with conservative management (preconservative score: 8.69 ± 1.09; postconservative score at 3 weeks: 6.33 ± 1.68). At 3 months, the VAS scores postconservative management in patients who did not give consent for surgical management were 7.32 ± 1.54, while the postsurgical scores were 2.94 ± 1.24.

## Discussion

The SP is embryologically derived from Reichert's cartilage, which is subdivided into four segments: tympanohyal, stylohyal (major part of SP), ceratohyal, and hypohyal. 13 14 Eagle divided his syndrome into two categories: classic and SP, and carotid artery syndrome. 10 Various theories are reported in literature to explain the etiology of Eagle's syndrome. The ones that gained popularity are the hyperplastic reaction, in which stimulation by pharyngeal trauma causes stylohyoid ligament ossification; the metaplastic reaction, in which cells in the styloid ligament undergo metaplastic alterations following a traumatic stimulus inducing partial or total ossification; and the anatomic variation, in which the elongation is attributed to anatomical variance rather than SP ossification. 9

In our series, the most common symptoms reported are recurrent oropharyngeal pain (67.8%), chronic neck pain (49%), foreign body sensation in throat (48.6%), and facial pain (45.2%). The less commonly reported symptoms include aggravation of symptoms when turning on affected side, headache, otalgia, and tinnitus. This presentation is similar to other studies reported in the literature. 12 Patients usually visit multiple doctors for this varied presentation of symptoms. Correct identification requires complete history taking, clinical examination, and radio-imaging, all of which can aid in appropriate diagnosis and treatment.

The mean length of SP in group 1 was 4.07 ± 0.64 cm on right and 4.02 ± 0.8 cm on left side; in group 2, the values were 2.36 ± 0.56 cm on the right and 2.28 ± 0.46 cm on left side. There is a statistically significant difference in length of SP between groups. These findings are similar to the studies by Okur et al., Yavuz et al., and Andrei et al., where the length of SP in stylalgia cases was between 3 and 6 cms. 3 6 15 There was no significant difference between length of right and left SP in cases/controls, between male/female patients and age groups. These findings are comparable to other studies. 3 6 16

Elongated SP can also be an incidental finding in many patients. Its presence alone might not produce all the symptoms of stylalgia. 6 8 9 Direction of elongation combined with abnormal angulation could cause irritation to the pharyngeal mucosa resulting in pain and foreign body sensation. Compression of various vital structures surrounding the SP could also result in varied symptoms. 4 16 Rare complications like syncope, transient ischemic attacks, cerebrovascular ischemia, carotid artery aneurysm, vagal cardiac inhibition leading to sudden death have also been reported. 17 18

Yavuz et al., in his study on 30 patients, reported that there was no difference between medial angulation of SP between cases and controls where as significant difference was noted in length and anterior angulation. 6 In a study done by Okur et al. where the angulation of SP was measured on 3D CT, the mean medial angulation was 22.60 ± 4.0 and 22.60 ± 4.5° on right and left sides, respectively. But, in the same group, there was no notable difference in anterior angulation, 3 unlike the present case series, where both anterior and medial angulations had a statistically significant difference within each group.

Rathva et al., in the study on morphology of normal and elongated SP found a marked variation in length, as well as anterior and medial angulations between the same. 5 Their anterior angulation was in the range of 0 to 57°, which is similar to our study. However, the medial angulation range was 0 to 62°, whereas in this study it ranged between 16 and 28°. It is important to note that Rathva et al.'s study measured angulations on the digital images of dry skulls. 5

The comparison of angulation parameters in our study revealed statistically significant differences in both anterior and medial angulations. This aligns with the findings by Rathva et al., 5 who demonstrated high variability in anterior (0–57°) and medial angulation (0–62°) in dry skulls. However, our values were measured in living subjects using X-ray imaging, which reflects more clinically relevant data. In contrast, Yavuz et al. 6 reported no significant difference in medial angulation, suggesting that differences in imaging modality or population demographics might account for this disparity. Furthermore, Okur et al., 3 using 3D-CT scans, found medial angulations of 22.6° bilaterally, which closely matches our findings for group 1 (mean: 21.28°). However, they did not report significant anterior angulation differences, unlike in our data. This suggests that both parameters might need to be considered together in clinical evaluations.

Most of the morphological variations reported in literature till date were on dry skulls, 5 8 11 and if reported in patients/dry skulls the variations were measured on 3D-CT. 3 7 12 16 19 Very few case series were done using X-Ray imaging. 6 12 In our series, we have put an effort to emphasize the usage of cost effective and easily available imaging technique for detecting the abnormal morphology of SP. As per the study, statistically remarkable differences were observed in the medial and anterior angulation of SP between cases and controls. This could be the reason for varied presentation, as medial angulation can cause mucosal irritation in tonsillar fossa and anterior angulation can cause irritation and pressure symptoms.

Stylalgia symptoms overlap with many other pathological conditions, which is why its diagnosis is usually missed. Some of the differential diagnosis for Eagle's syndrome include temporomandibular joint dysfunction, myofascial pain, and unerupted molar tooth, as well as trigeminal, glossopharyngeal, laryngeal, occipital, and sphenopalatine neuralgia. 10 11 20 Glossopharyngeal neuralgia is differentiated from Eagle's syndrome by its characteristic short duration of sharp, jabbing pain, similar to electric shocks.

Conservative management includes treatment with anticonvulsant and antidepressant medications. Our patients received pregabalin, amitriptyline, and tramadol. These medications were chosen based on their multimodal mechanisms of action. Pregabalin helps in modulating calcium channel activity to reduce neuropathic excitability. Amitriptyline increases descending pain inhibition through serotonergic and noradrenergic pathways. Tramadol acts on μ-opioid receptors and helps in inhibition of serotonin and norepinephrine reuptake. Together, both the neuropathic and nociceptive components of stylalgia are targeted. As per the literature, other treatment options include nerve blocks, local anesthetic or steroid injections in the tonsillar fossa, and physiotherapy. 21 22 23

Han et al., 21 in a case report on nonsurgical management of Eagle's syndrome, used a combination of gabapentin, tramadol hydrochloride, tianeptine, triamcinolone with mepivacaine injection into tonsillar fossa, and stellate block. Gradual improvement of symptoms was seen after 2 weeks. 21 In a case report by Malik et al., treatment with pregabalin and carbamazepine showed successful results in relief of symptoms. 22 The VAS scores showed a significant relief in symptoms after 3 weeks of conservative management. However, this decrease was lower when compared with patients who underwent surgical management.

Surgical management by styloidectomy was done in 62% of the patients in this case series. Symptomatic relief was noted in all the patients by comparing the VAS before and at 3 weeks and 3 months after the surgery. Naik et al., in a study done on tonsillo-styloidectomy in Eagle's syndrome, mentioned a definitive relief in symptoms of stylalgia after styloidectomy. 24 Many case reports till date showed the effectiveness of styloidectomy in alleviating pain. 25 26 27 A long term follow-up is necessary to assess recurrence of symptoms. To date, no patients complained of recurrence of symptoms in this series.

### Limitations and Recommendations

Sample size and lack of long-term follow-up were the limitations of this study. It is recommended that a large sample size with inputs from various institutes across the world using the same technique will give us more appropriate data. The addition of long-term follow-ups to data collection will improve the assessment of symptomatic outcomes following conservative or surgical management.

## Conclusion

Symptomatic elongated SP is a complex condition for many patients. Precise identification of this entity will aid in early recovery from symptoms. Diagnosing elongated SP can be done with X-ray imaging, which is cost-effective, less time consuming, easily available, and more accessible to different economic backgrounds.

In this series, statistically significant difference was noted in length, along with medial and anterior angulations in stylalgia cases when compared with controls, indicating the elongation and abnormal angulation can result in varied symptoms of this condition. Though conservative management with anticonvulsant and antidepressant drugs provides pain relief, it's considered temporary. Patients in this series reported better improvement in VAS scores following surgical management.

## References

[1] Abuhaimed A K Alvarez R Menezes R G Anatomy, Head and Neck, Styloid Process Treasure Island, FL StatPearls Publishing 2025Available from:https://www.ncbi.nlm.nih.gov/books/NBK540975/31082019

[2] Mudry A From the stylet of the temple to the tongue in so-called Riolan's bouquet Eur Ann Otorhinolaryngol Head Neck Dis 2020 137 04 347 348 10.1016/j.anorl.2020.01.020 32192900

[3] Okur A Ozkırış M Serin H I Is there a relationship between symptoms of patients and tomographic characteristics of styloid process? Surg Radiol Anat 2014 36 07 627 632 10.1007/s00276-013-1213-2 24158351

[4] Czako L Simko K Thurzo A Galis B Varga I The Syndrome of Elongated Styloid Process, the Eagle's Syndrome-From Anatomical, Evolutionary and Embryological Backgrounds to 3D Printing and Personalized Surgery Planning. Report of Five Cases Medicina (Kaunas) 2020 56 09 458 10.3390/medicina56090458 32916813 PMC7558969

[5] Rathva A Kubavat D M Nagar S K Study of styloid process: anatomical variations in length, angulation and distance between the two styloid processes Int J Recent Trends Sci Technol 2013 8 02 109 112

[6] Yavuz H Caylakli F Yildirim T Ozluoglu L N Angulation of the styloid process in Eagle's syndrome Eur Arch Otorhinolaryngol 2008 265 11 1393 1396 10.1007/s00405-008-0686-9 18427825

[7] Gözil R Yener N Calgüner E Araç M Tunç E Bahcelioğlu M Morphological characteristics of styloid process evaluated by computerized axial tomography Ann Anat 2001 183 06 527 535 10.1016/S0940-9602(01)80060-1 11766524

[8] Patil S Ghosh S Vasudeva N Morphometric study of the styloid process of temporal bone J Clin Diagn Res 2014 8 09 AC04 AC06 10.7860/JCDR/2014/9419.4867 PMC4225865 25386413

[9] Piagkou M Anagnostopoulou S Kouladouros K Piagkos G Eagle's syndrome: a review of the literature Clin Anat 2009 22 05 545 558 10.1002/ca.20804 19418452

[10] Eagle W Elongated styloid processes: Report of Two Cases Arch Otolaryngol Head Neck Surg 1937 25 05 584 587 10.1001/archotol.1937.00650010656008

[11] Vadgaonkar R Murlimanju B V Prabhu L V Morphological study of styloid process of the temporal bone and its clinical implications Anat Cell Biol 2015 48 03 195 200 10.5115/acb.2015.48.3.195 26417479 PMC4582162

[12] Mazzetto M O Andrade K M Magri L V Rodrigues C A Watanabe P C Anterior and medial angulations of the styloid process in subjects with TMD: clinical and radiographic findings Braz Dent J 2013 24 01 80 84 10.1590/0103-6440201302126 23657419

[13] Cawich S O Gardner M Shetty R Harding H E A post-mortem study of elongated styloid processes in a Jamaican populationInternet J Biol Anthropol 2008;3(01). Available from:https://ispub.com/IJBA/3/1/4152

[14] Lorman J G Biggs J R The Eagle syndrome AJR Am J Roentgenol 1983 140 05 881 882 10.2214/ajr.140.5.881 6404147

[15] Andrei F Motoc A GM Didilescu A C Rusu M C A 3D cone beam computed tomography study of the styloid process of the temporal bone Folia Morphol (Warsz) 2013 72 01 29 35 10.5603/fm.2013.0005 23749708

[16] Onbas O Kantarci M Murat Karasen R Angulation, length, and morphology of the styloid process of the temporal bone analyzed by multidetector computed tomography Acta Radiol 2005 46 08 881 886 10.1080/02841850500335085 16392614

[17] Dao A Karnezis S Lane J S III Fujitani R M Saremi F Eagle syndrome presenting with external carotid artery pseudoaneurysm Emerg Radiol 2011 18 03 263 265 10.1007/s10140-010-0930-7 21213007 PMC3095808

[18] Todo T Alexander M Stokol C Lyden P Braunstein G Gewertz B Eagle syndrome revisited: cerebrovascular complications Ann Vasc Surg 2012 26 05 7290 7.29E7 10.1016/j.avsg.2011.12.005 22664285

[19] Oztunç H Evlice B Tatli U Evlice A Cone-beam computed tomographic evaluation of styloid process: a retrospective study of 208 patients with orofacial pain Head Face Med 2014 10 01 5 10.1186/1746-160x-10-5 24528515 PMC3943457

[20] Mendelsohn A H Berke G S Chhetri D K Heterogeneity in the clinical presentation of Eagle's syndrome Otolaryngol Head Neck Surg 2006 134 03 389 393 10.1016/j.otohns.2005.10.046 16500433

[21] Han M K Kim D W Yang J Y Non Surgical Treatment of Eagle's Syndrome - A Case Report -Korean J Pain 2013 26 02 169 172 10.3344/kjp.2013.26.2.169 23614080 PMC3629345

[22] Malik Y Dar J A Almadani A A Seizures with an atypical aetiology in an elderly patient: Eagle's syndrome–how does one treat it? BMJ Case Rep 2015 2015 bcr2014206136 10.1136/bcr-2014-206136 PMC4680275 26604239

[23] Taheri A Firouzi-Marani S Khoshbin M Nonsurgical treatment of stylohyoid (Eagle) syndrome: a case report J Korean Assoc Oral Maxillofac Surg 2014 40 05 246 249 10.5125/jkaoms.2014.40.5.246 25368838 PMC4217270

[24] Naik S M Naik S S Tonsillo-Styloidectomy for Eagle's Syndrome: A Review of 15 Cases in KVG Medical College Sullia Oman Med J 2011 26 02 122 126 10.5001/omj.2011.30 22043398 PMC3191668

[25] Walli A K Thorawade V P Parelkar K Nagle S Kulsange K L Intraoral Styloidectomy in Eagle's Syndrome-A Risky and Infrequently Performed Approach J Clin Diagn Res 2018 12 01 MD01 MD02 10.7860/jcdr/2018/27339.11085

[26] Khandelwal S Hada Y S Harsh A Eagle's syndrome – A case report and review of the literature Saudi Dent J 2011 23 04 211 215 10.1016/j.sdentj.2010.10.006 23960519 PMC3723259

[27] Bahgat M Bahgat Y Bahgat A Eagle's syndrome, a rare cause of neck pain BMJ Case Rep 2012 2012 bcr2012006278 10.1136/bcr-2012-006278 PMC4543270 22843755

